# Determining the timing of respiratory syncytial virus (RSV) epidemics: a systematic review, 2016 to 2021; method categorisation and identification of influencing factors 

**DOI:** 10.2807/1560-7917.ES.2024.29.5.2300244

**Published:** 2024-02-01

**Authors:** Lisa Staadegaard, Michel Dückers, Jojanneke van Summeren, Rob van Gameren, Clarisse Demont, Mathieu Bangert, You Li, Jean-Sebastien Casalegno, Saverio Caini, John Paget

**Affiliations:** 1 Netherlands Institute for Health Services Research (Nivel), Utrecht, The Netherlands; 2 Faculty of Behavioural and Social Sciences, University of Groningen, Groningen, The Netherlands; 3 ARQ National Psychotrauma Centre, Diemen, The Netherlands; 4 Sanofi Pasteur, Lyon, France; 5 National Vaccine Innovation Platform, School of Public Health, Nanjing Medical University, Nanjing, China; 6 Centre for Global Health, Usher Institute, University of Edinburgh, Edinburgh, Scotland, United Kingdom; 7 Hospices Civils de Lyon; Hôpital de la Croix-Rousse; Centre de Biologie Nord; Institut des Agents Infectieux; Laboratoire de Virologie, Lyon; France

## Abstract

**Background:**

There is currently no standardised approach to estimate respiratory syncytial virus (RSV) epidemics’ timing (or seasonality), a critical information for their effective prevention and control.

**Aim:**

We aimed to provide an overview of methods to define RSV seasonality and identify factors supporting method choice or interpretation/comparison of seasonal estimates.

**Methods:**

We systematically searched PubMed and Embase (2016–2021) for studies using quantitative approaches to determine the start and end of RSV epidemics. Studies’ features (data-collection purpose, location, regional/(sub)national scope), methods, and assessment characteristics (case definitions, sampled population’s age, in/outpatient status, setting, diagnostics) were extracted. Methods were categorised by their need of a denominator (i.e. numbers of specimens tested) and their retrospective vs real-time application. Factors worth considering when choosing methods and assessing seasonal estimates were sought by analysing studies.

**Results:**

We included 32 articles presenting 49 seasonality estimates (18 thereof through the 10% positivity threshold method). Methods were classified into eight categories, two requiring a denominator (1 retrospective; 1 real-time) and six not (3 retrospective; 3 real-time). A wide range of assessment characteristics was observed. Several studies showed that seasonality estimates varied when methods differed, or data with dissimilar assessment characteristics were employed. Five factors (comprising study purpose, application time, assessment characteristics, healthcare system and policies, and context) were identified that could support method choice and result interpretation.

**Conclusion:**

Methods and assessment characteristics used to define RSV seasonality are heterogeneous. Our categorisation of methods and proposed framework of factors may assist in choosing RSV seasonality methods and interpretating results.

**Key public health message**

**What did you want to address in this study?**

Determining the start, end or duration of respiratory syncytial virus (RSV) epidemics is key to implementing timely public health measures to control such epidemics. Nevertheless, the many ways to calculate these metrics complicate the interpretation and comparison of findings. We wanted to gain an overview of methods to define the RSV season, to organise them, and develop a framework for choosing methods and interpreting their outcome.

**What have we learnt from this study?**

By performing a systematic literature review looking at both the characteristics of methods and the context in which these are applied, we were able to show a large heterogeneity in the current estimations of timing of RSV epidemics. We organised the methods that we found into eight broad categories and provided a framework of five factors that may help to choose among estimation methods as well as understand and contextualise their results.

**What are the implications of your findings for public health?**

In categorising methods and devising a framework, this study contributes to a more rigorous application and interpretation of seasonality estimates. This should support effective and efficient implementation of future public health measures aiming to lower the burden of RSV infections. In addition, our findings may more widely apply to other respiratory infections like those by severe acute respiratory syndrome coronavirus 2 (SARS-CoV-2).

## Introduction

Respiratory syncytial virus (RSV) is a common respiratory pathogen that causes infections of the respiratory tract and results in seasonal epidemics in many areas of the world [1]. In 2019, it was estimated that RSV infections in children below the age of 5 years resulted in ca 3.6 million hospital admissions globally [2]. Nevertheless, infections affect all age groups, with reinfections possible throughout life [3].

Timely prevention and control interventions are likely to reduce the substantial burden posed by RSV on both individuals affected by the virus and on the healthcare system [4,5]. Until recently the only available prophylaxis was palivizumab, a monoclonal antibody (mAb), but as of September 2022 the European Medicines Agency (EMA) gave market authorisation for nirsevimab, another mAb [6]. Whereas palivizumab is only recommended for high-risk infants (i.e. born prematurely, suffering from congenital heart disease) and requires monthly administration during the RSV season, nirsevimab has been approved for all infants at the beginning of their first RSV season (or as soon as possible after birth, if born during the RSV season) and a single dose protects them for ca 5 months [6,7]. Other preventative measures are in various stages of clinical development [8].

Importantly, the monthly administration of palivizumab puts a considerable strain on infants and comes at a high cost, with a United States (US)-based study from 2011 finding that the mean payment per dose may exceed 2,000 dollars (ca. 1,800 euro) [9]. Although nirsevimab has the potential to be more cost-effective, both prevention methods have a limited duration of protection and are thus time-sensitive [10]. As a consequence, to be effective, prevention strategies should be timely, and this is largely dependent on a thorough understanding of the timing (also referred to as seasonality) of RSV epidemics.

**Figure 1:**
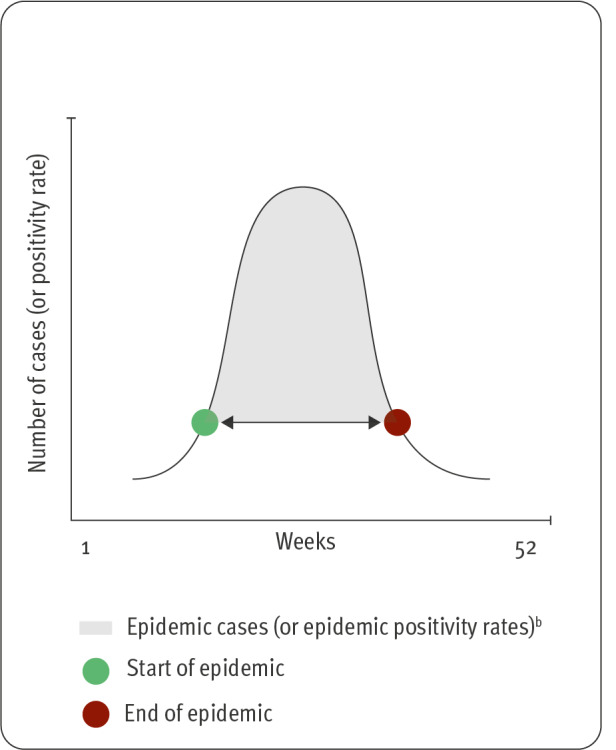
Parameters used to characterise a respiratory syncytial virus epidemic (n = 3 parameters)^a^

^a^ The three parameters are the start (green), end (red) and capture rate (grey).

^b^ The shaded area (grey) represents either the numbers of cases belonging to the epidemic or the positivity rates during the epidemic. These values enable to calculate the capture rate (i.e. the proportion of annual cases who fall within the epidemic). Annual cases in the denominator of the capture rate are the number of cases detected by the surveillance system in a period of a year.

A number of studies published between the end of the 2010s and the beginning of the 2020s have shown that while timing of RSV epidemics can vary in (sub)tropical areas [11], RSV activity mostly peaks in winter months in places with a temperate climate [1,12,13]. Still, unexplained changes in seasonality can occur anywhere, as exemplified by striking shifts in the start and end weeks of RSV epidemics that were observed during – and influenced by – the COVID-19 pandemic [14-19]. In this regard, continued collection of (sub)national data on RSV infections, as well as their analysis remains critical to define the start, end or ‘capture rate’ (i.e. proportion of annual cases who belong to the epidemic) of RSV epidemics, as this allows to apply mitigation and control measures when most needed (Figure 1).

The arrow indicates the duration of the epidemic.

Currently a wide variety of methods are reported in the literature to define the start and end of RSV epidemics. One of these, which is commonly employed by the US Center for Disease Control and Prevention (CDC), is the ‘10% positivity threshold’, a method that defines the RSV epidemic onset week, as the first, or the first of consecutive weeks, with a percentage of positive RSV tests (among all tests performed) exceeding 10% [20]. The World Health Organization (WHO) recommends using the Moving Epidemic Method (MEM) to estimate seasonality of influenza epidemics [21], but no recommendation exists for RSV to date. A paper published in 2021 based on a workshop attended by European RSV experts stated that each country should apply the best calculation method “*according to availability of the data and local circumstances*” [11]. 

Remarkably, the choice for the estimation method, possibly driven by data availability and local circumstances, may impact the eventual definition of the timing of the RSV epidemic. For example, a study in Slovenia comparing four different methods to define an RSV epidemic showed that while these found a similar timing of the peak, epidemic duration estimates differed by up to 7 weeks, with implications for (potential) prevention activities [13]. Despite the existence of other investigations comparing several methods with each other [12,22,23], there is currently, to the best of our knowledge, no comprehensive overview of methods used to estimate the timing of RSV epidemics, the context in which they are applied, and how this could potentially impact the outcome.

Therefore, our objective was to perform a literature review of such methods and to simultaneously record some characteristics that seasonality determination rely on. A secondary aim was to classify the methods and characterise the context and underlying data of studies found, in order to develop a framework of categories and factors that might help interpreting seasonal estimates and choosing methods in future investigations.

## Methods

### Search strategy and study inclusion

We systematically searched PubMed and Embase using terms for ‘RSV’ and ‘seasonality’ published between January 2016 and February 2021. The full search terms are described in the Supplementary Material. Titles and abstracts were first screened by two independent researchers (LS and JvS) after which full text publications were retrieved and reviewed (LS and SC). Full text articles were checked for relevant references (also published between January 2016 and February 2021) and publications identified this way, fulfilling the below criteria were also included. Publications were eligible for inclusion if they provided methods to estimate the timing (start, end or duration) of human RSV epidemics on subnational, national or regional level including abstracts or conference proceedings. All included full-text articles were peer reviewed. No language restrictions were used. Commentaries or reviews that did not use a quantitative method to provide new estimates were excluded.

### Data extraction

Data from studies included in the review were extracted with the aim of improving our understanding of RSV seasonality estimates and of identifying factors that might influence estimations and complicate comparison across studies.

First, we recorded details on the features of the study (e.g. date of publication, study purpose, location, WHO region of provenance and geographical scope) for each included publication.

Second, certain attributes of the estimation method were extracted. This information pertained to the title of the method applied and the rules that were used to determine the exact start and end of RSV epidemics. It included the type of threshold used and the number of gap weeks that were allowed. Here, ‘gap’ weeks refer to the number of weeks the number of cases or the positivity rate is allowed to be below the threshold (or missing) as to still be included as part of the epidemic period. Attributes of the methods also included the timing of the analysis (i.e. if the method was used retrospectively, in real time or prospectively), if the method required the data to be transformed (e.g. moving average or wavelet transformation) and the type of data required for the method (numerator or denominator). 

Finally, we extracted information on assessment characteristics, such as the period of data collection (i.e. year-round or not), the case definition and testing practices used, the age of the sampled population, and the setting in which cases were enrolled (inpatient or outpatient). 

Data were extracted by LS and reviewed by SC or RvG; differences were resolved by consensus and where necessary a third researcher was consulted.

### Categorisation of methods

Identified methods were categorised based on (i) the data required to perform the analysis (numerator or denominator) and (ii) the time frame of the analysis (real-time or retrospective).

## Results

**Figure 2:**
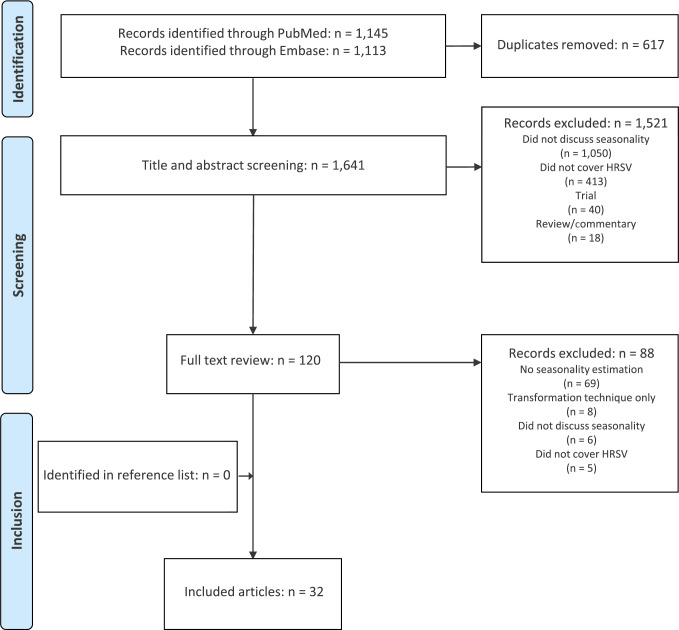
Flowchart of the selection of studies in a systematic review on methods to determine seasonality of respiratory syncytial virus epidemics, 2016−2021 (n = 2,258 articles screened)

Of the 2,258 publications identified through our search, 120 underwent full text review of which 32 met our inclusion criteria (Figure 2). No relevant additional articles were identified by searching the reference lists of included articles.

HRSV: human respiratory syncytial virus.

### Details of the included studies

#### Study characteristics

The majority of articles included in our study used national data (n = 16) followed by subnational (e.g. province) (n = 11) and regional data (e.g. WHO region) (n = 5) (Table 1). Most articles came from the WHO Region of the Americas (n = 15) and the aim of data collection was primarily for regular surveillance (n = 20, Table 1), or for other purposes (n = 12) such as reviews, claim or inpatient databases or solely for the purpose of their study.

**Table 1:** Overview of study, method, and assessment characteristics described in articles included in a systematic review on approaches to determine seasonality of respiratory syncytial virus epidemics, 2016−2021 (n = 32 articles)

Elements considered			Number of analyses	Total number of analyses in the studies	%	Citations of the studies
**Study features**	**Geographical scope**	Regional	5	32	16%	[1,25,27,40,41]
		National	16	32	50%	[12,13,20,22,23,26,42-51]
		Subnational	11	32	34%	[52-62]
	**Provenance of the study (WHO region/global)**	African	1	32	3%	[59]
		Americas	15	32	47%	[20,22,23,26,42,44,46-50,54,56,60,61]
		European	10	32	31%	[12,13,25,41,43,45,53,55,57,62]
		Western Pacific	2	32	6%	[51,52]
		South-East Asian	1	32	3%	[58]
		Global	3	32	9%	[1,27,40]
	**Purpose of data collection**	Surveillance	20	32	63%	[12,13,20,22,23,25,26,40,41,43,44,46-50,55,56,59,62]
		Other	12	32	38%	[1,27,42,45,51-54,57,58,60,61]
**Attributes of the analysis^a^**	**Timing of analysis**	Retrospective	30	32	94%	[1,12,13,20,22,23,25-27,40-42,44,45,47-62]
		Real-time	1	32	3%	[43]
		Prospective	1	32	3%	[46]
	**Transformation method**	Yes	10	32	31%	[12,20,22,40,42,43,46,52,56,59]
		No	22	32	69%	[1,13,23,25-27,41,44,45,47-51,53-55,57,58,60-62]
	**Methods^b^**	Percentage positive threshold: 10%	18	49	37%	[13,22,23,27,40,44,46-48,52,54,55,57,62]
		Percentage positive threshold: various others	7	49	14%	[13,22,23,49]
		Number of detections threshold: various	5	49	10%	[12,20,22,42]
		Percentage of detections threshold: 1.2%	3	49	6%	[12,13,25]
		Percentage of detections: various others	4	49	8%	[41,50,53,58]
		Mean detections threshold: 60% threshold	2	49	4%	[13,60]
		Mean detections threshold: various others	2	49	4%	[45,61]
		Mean % positive threshold	3	49	6%	[26,51,59]
		Average annual percentage	1	49	2%	[1]
		Change point analysis	1	49	2%	[56]
		Moving epidemic method (MEM)	3	49	6%	[12,13,43]
**Assessment characteristics**	**Year-round data collection**	Yes	20	32	63%	[12,13,20,23,26,40,43,45,47,49,51-53,56-62]
		No	3	32	9%	[44,48,55]
		Unknown	9	32	28%	[1,22,25,27,41,42,46,50,54]
	**Age group sampled**	All ages	13	32	41%	[12,13,22,23,26,40,44,46,48,55,57-59]
		Children (< 18 years old)	10	32	31%	[43,45,47,51-54,60-62]
		Unknown	9	32	28%	[1,20,25,27,41,42,49,50,56]
	**Setting**	Inpatient	9	32	28%	[42,45,47,50-54,57]
		Outpatient	3	32	9%	[43,55,58]
		Mix	10	32	31%	[12,13,20,26,40,41,48,56,59,62]
		Unknown	10	32	31%	[1,22,23,25,27,44,46,49,60,61]
	**Case definition for test inclusion^c^**	ALRI	2	32	6%	[47,54]
		ARI	1	32	3%	[57]
		ILI	2	32	6%	[55,58]
		SARI	0	32	0%	No study
		ICD codes for RSV	3	32	9%	[42,45,51]
		Other/mix^d^	10	32	31%	[12,25,26,40,43,50,52,53,59,62]
		Unknown	14	32	44%	[1,13,20,22,23,27,41,44,46,48,49,56,60,61]
	**Diagnostics**	Antigen	4	32	13%	[23,26,44,48]
		PCR	12	32	38%	[12,13,20,22,40,49,50,52,53,55,57,58]
		Other/mix	9	32	28%	[25,43,46,47,54,56,59,61,62]
		Unknown	7	32	22%	[1,27,41,42,45,51,60]

ALRI: acute lower respiratory infections; ARI: acute respiratory infection; ICD: International Classification of Diseases; ILI: influenza-like illness; RSV: respiratory syncytial virus; SARI: severe acute respiratory infection; WHO: World Health Organization.

^a^ Four studies [12,13,22,23] compared two or more methods with each other.

^b^ Number of overall methods (n = 49) is higher than the total of articles (n = 32) included as some studies compared several methods or implemented one method more than once with different assessment characteristics.

^c^ For respiratory infections (e.g. ARI and ILI), articles most commonly cited World Health Organization definitions, but not consistently.

^d^ This includes studies with a mix of any combination of either/and/or ARI.ILI, SARI, ALRI cases.

#### Characteristics of the methods used

Of the included studies, 30 performed their analysis retrospectively and 10 transformed the data (e.g. moving average or wavelet transformation) before applying an estimation method. Most studies applied a single estimation technique (n = 28), with four studies comparing the results of more than one method (Table 1). Eighteen estimates were based on the 10% positivity threshold, while seven estimates used a different percentage positivity threshold (e.g. 3, 5 or 7%). Other studies applied estimation methods that used a percentage (e.g. 1.2, 5 or 10%) of the total cases as a threshold (n = 7). Across studies, even when applying the same method, the exact application varied with differences in the number of gap weeks that were allowed as well as the minimum number of specimens tested (Supplementary Table 1). 

#### Assessment characteristics

Most studies (n = 20) collected data all year round. Thirteen studies included data that sampled all age categories and 10 only sampled children (< 18 years old). Most often, patients emerged from a mixed setting (meaning both inpatients and outpatients, n = 10) or from an unknown setting (n = 10). The case definition for test inclusion (e.g. acute lower respiratory infections (ALRI) (n = 2)) in the papers varied and for most studies the case definition for inclusion was unknown (n = 14) or mixed (i.e. any combination of either/and/or acute respiratory infection (ARI), influenza-like illness (ILI), severe ARI (SARI), ALRI (n = 10)). Cases were mostly confirmed using PCR (n = 12) followed by nine studies using a mix, or other methods (e.g. combination of antigen and PCR) and four studies using antigen testing only (Table 1). Further details on extracted information on study level can be found in Supplementary Table 1.

### Categorisation of seasonality estimation methods

Methods were categorised based on the data needs (e.g. numerator only or denominator) as well as the time frame in which they could be applied (e.g. retrospective or real-time). Though we found heterogeneity in the application of several methods (e.g. minimum number of specimens tested, see Supplementary Table 1), we organised the identified methods into eight broad categories (method I–VIII; Table 2).

**Table 2:** Categorisation of seasonality methods included in the systematic review and their definition of the start and end of the respiratory syncytial virus (RSV) season, 2016−2021 (n = 32 articles)

Data requirement	Timing	Name	Start	End	Source
**Denominator**	Real-time (potential)	I: % positivity threshold	1^st^ or 1^st^ of 2 consecutive weeks when positivity exceeds threshold (thresholds used; 3,5, 7 or 10%)	Last or last of 2 consecutive weeks with positivity above threshold	[13,22,23,27,40,44,47,48,52,54,55,57,62]
		II: MEM	1^st^ week when curve exceeds the epidemic threshold (based on historical surveillance data)	1^st^ week when curve is below the post-epidemic threshold (based on historical surveillance data)	[12,13,43]
	Retrospective	III: mean positivity threshold	Various (e.g. 1^st^ of 3 consecutive weeks or 1^st^ week with percentage positive exceeding mean positivity threshold)	Various (e.g. 3^rd^ of 3 consecutive weeks that occur at least 5 weeks following season onset, where the percentage positive is below the mean positivity threshold)	[26,51,59]
**Numerator**	Real-time (potential)	IV: Number of detections threshold	Various (e.g. 1^st^ week with at least 20 detections, 3 consecutive weeks with at least 6 RSV hospitalisations per week, RS10, 10-fold baseline)	Various (e.g. last week with at least 20 detections or 3 weeks with 6 or more RSV hospitalisations consecutively)	[12,20,22,58]
		II: MEM	1^st^ week when curve exceeds the epidemic threshold (based on historical surveillance data)	1^st^ week when curve is below the post-epidemic threshold (based on historical surveillance data)	[12,13,43]
		V: change point analysis	No formal definition, modelled via change point analysis	No formal definition, modelled via change point analysis	[56]
	Retrospective	VI: % of detections threshold	Various (e.g. 1^st^ week RSV detections exceed 1.2% of total RSV positive specimens)	Various (e.g. last week RSV detections exceed 1.2% of total RSV positive specimens)	[12,13,25,50,53,58]
		VII: mean detections threshold	Various (e.g. above the average weekly number of cases, 60% threshold)	Various (e.g. below the average weekly number of cases, 60% threshold)	[13,45,60,61]
		VIII: AAP	1^st^ month of the longest period of consecutive months to be included in the sorted AAP 75%	Last month of the longest period of consecutive months to be included in the sorted AAP 75%	[1]

AAP: average annual percentage. MEM: moving epidemic method. RSV: respiratory syncytial virus; RS10: retrospective slope 10.

#### Data requirement

The methods we identified could be categorised according to whether they were restricted by the availability of a denominator; the denominator being the number of specimens tested. Two of the methods (percentage (I) and mean positivity threshold (III), Table 2) require a denominator as they rely on the positivity rate to set a threshold for the beginning and end of the RSV season. One (I) uses a predetermined positivity rate as a threshold (e.g. 10%), the other (III) the average positivity rate of the season in question. Six types of methods can be applied either in the absence of a denominator, or in cases where the numerator is deemed more reliable. These are the MEM (II), the number (IV), percentage (VI) or mean (VII) of detections threshold, as well as the change point analysis (V), and the average annual percentage (VIII). The MEM (II) is the only one that can be used both with or without the availability of a denominator.

#### Time frame

The methods we identified could also be categorised into those with real-time potential (I, II, IV and V; n = 4) and those that can solely be used retrospectively (III and VI−VIII; n = 4). The latter can only be applied once the season is over as these methods rely on either the total number of cases or positivity rate across that specific season, whereas the former allow the tracking of the epidemic as it unfolds as they solely rely on the current case count or positivity rate. Nevertheless, the methods categorised as ‘real-time (potential)’ can also be applied retrospectively and were often used as such in the studies we identified (Supplementary Table 1). Noteworthy is that although MEM can be used to identify RSV seasonality in real time, it does require data from previous seasons. The method is slightly different from the rest as its main purpose is the calculation of a threshold as an early warning for future epidemics and it also allows the assessment of the intensity of the epidemic [12,13]. 

### Considerations for the choice of a seasonality estimation method and outcome interpretation

**Figure 3:**
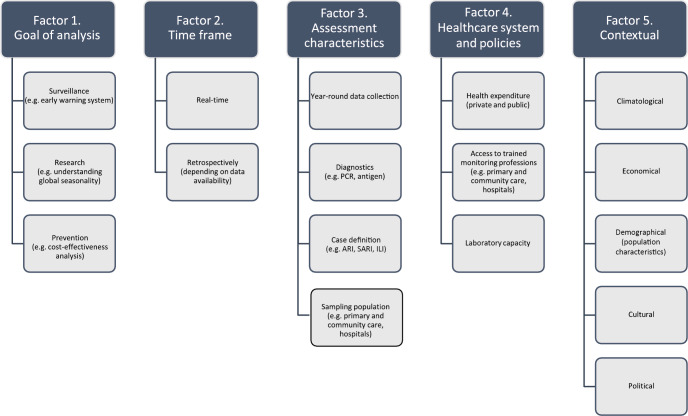
Factors to be considered when choosing a method to determine the timing of a respiratory syncytial virus (RSV) epidemic, systematic review, 2016−2021 (n = 32 articles included)

Based on the literature review and the extracted data, we established five broad factors that should be considered when choosing a seasonality estimation method and interpreting the results (Figure 3).

ARI: acute respiratory infection; ILI: influenza-like illness; SARI: severe acute respiratory infection; PCR: polymerase chain reaction.

#### Goal of the analysis

The first point to be considered, in particular when choosing a method, is the ‘goal of the analysis’. Most articles included in the current review described their analyses solely as part of regular surveillance reporting (n = 20; Table 1). For early warning purposes, a method applied in real-time would be necessary. The MEM, which has the advantage of being able to prospectively determine an epidemic threshold as well could be a suitable method here, possibly resulting in more focused diagnostics and treatment during an RSV epidemic [12]. However, objectives of studies may vary, especially if the seasonality estimate is required for research purposes. Examples include identifying trends or comparing seasonality estimates using a multi-country dataset [1,19,20]. As mentioned, if the aim is to provide insight into the intensity of the epidemic, the MEM could also be a suitable method [12]. Another goal is to provide an analysis for the implementation of an (cost-)effective prevention measure.

#### Time frame

The second factor, also especially relevant when choosing a method, is the ‘time frame’: whether the method has the potential for an analysis to be performed in real-time as opposed to solely retrospectively. Importantly, most studies included in the review had a retrospective focus (30 of 32 Table 1), but the ‘time frame’ limits the choice for a seasonality estimation method (Table 2).

#### Assessment characteristics

The ‘assessment characteristics’ constitute the third factor that should be considered both when choosing a method and interpreting results. The ‘assessment characteristics’ cover a diverse range of issues such as whether RSV surveillance data are collected year-round or the type of diagnostic method that is used (e.g. PCR vs antigen). In terms of choosing a method, when the data collection is limited to the winter season, the retrospective slope 10 (RS10) or 10-fold baseline approach, which require year-round surveillance, cannot be used [22]. In addition, unexpected early or long epidemics might not be identified using a limited surveillance period, which is a point to consider in interpreting results from any method based on this type of assessment.

For the diagnostic method, the US CDC found that PCR testing, as opposed to antigen testing, resulted in far lower positivity rates possibly reflecting differences in setting and use of both methods [22,23]. This means that the 10% positivity threshold method might be inappropriate for defining RSV seasonality in the case of PCR testing, and this threshold may need to be adapted to this type of testing; yet six of the 12 studies that used exclusively PCR (Table 1) applied a 10% threshold [22,23]. Regardless of this, PCR has a much better performance (sensitivity and specificity) than antigen tests, especially when the true RSV prevalence is low (e.g. during the epidemic shoulders) [24], and should be recommended as the method of choice whenever feasible. 

In addition, the included studies used a wide variety of criteria or sources to identify RSV cases. Studies employed different case definitions (e.g. ARI, extended SARI, or  ILI), and cases were ascertained from different types of care (e.g. inpatient or outpatient). These criteria/sources can affect seasonality estimates, as shown by two studies. The first, comparing RSV seasonality across Europe, differentiated between sentinel (primary) and non-sentinel (primary and/or hospital care facilities) surveillance data and showed small differences in the timing of RSV epidemics when comparing data from both sources [25]. The second, by Vos et al. found a different start and duration of the RSV season when comparing the use of data emerging from a hospitalised or community setting [12]. The authors speculate that this variation could have emerged due to differences between the populations included in both datasets (e.g. inclusion of a more vulnerable patient population or wider coverage).

#### Healthcare system and policies

The fourth factor to be considered, is the environment within which the data are collected: the ‘healthcare system and policies’ which cover issues like public and private health expenditure, access to general practitioners and hospitals, as well as laboratory capacity. This factor can be relevant to consider both when choosing a method or interpreting results. In terms of choosing a method, this may restrict the approach that can be used, but this also might have relevance when trying to understand the results of seasonality analysis. An example of this is found in the primary care surveillance in the Netherlands, where the proportion of samples positive for RSV has been increasing over time, possibly due to increasing awareness of RSV resulting in more selective sampling and consulting [12]. Another such example was found in Argentina, where the number of specimens tested dropped in 2011 as a result of a transition from paper to electronic records [26].

#### Contextual

Finally, the fifth factor is more general and concerns the overall context where the data are collected. Within this factor, the temperate vs sub-tropical or tropical latitude of areas affects the nature and availability of data or seasonality patterns. Similarly to factor 3 and 4, the context can be relevant when choosing a method as well as interpreting results. Whereas countries in temperate climates are likely to experience clear, one peak, RSV epidemics, countries in other climate zones may experience secondary peaks or even year-round RSV activity [1,27]. Some methods may be better suited to define these types of RSV seasonality. One example of this is the review by Li et al., where the average annual percentage (AAP) method was adjusted so that it could be used to identify secondary peaks in the number of RSV cases [1]. Social and economic (e.g. country income level) ‘contextual’ aspects are also important to bear in mind, as these could impact the (quality of) data and/or potentially bias results. Similarly, population density and age distribution (i.e. prevalence of risk groups like infants and those over 65 years old) have also been shown to affect data collection, as is supported by research on severe acute respiratory coronavirus 2 (SARS-CoV-2) [28].

## Discussion

For both healthcare providers and policy makers to timely respond to RSV epidemics, as well as to ensure the efficient implementation of costly and time sensitive interventions, the ability to adequately define the seasonality of RSV is critical. To this end, a variety of methods have been developed, and a few studies comparing the outcomes of some of these have shown that definitions of RSV epidemics could vary according to the estimation technique used [12,13,22,23]. Hence, in the absence of a standard way to assess seasonality [11], how to decide on a ‘correct’ estimation technique can be challenging, with in turn, potential consequences for timely informing healthcare services or raising public awareness. While previous work contrasting some methods and their outcomes has been of value [12,13,22,23], gaining a more comprehensive overview of existing methods, particularly recent ones, offers a future perspective of more extensive comparisons to support continued guidance. Therefore, we systematically reviewed PubMed and Embase for studies published between 2016 and 2021, which used quantitative approaches to determine the start and end of RSV epidemics.

Our investigation found a large range of methods used to describe seasonality, and also showed that these were not always consistent in their application (e.g. different number of gap weeks). Assessment characteristics, moreover, varied widely, which may complicate the interpretation of the studies’ outputs or the comparison of seasonality estimates across the literature or internationally. By examining the methods found, we could group them into eight broad categories based on the data needs (numerator only or denominator) and the time frame in which methods could be used (retrospective vs real-time). In addition, we identified five factors that can be considered when looking into a method to estimate RSV seasonality or interpreting its results.

One of these factors, which can have implications for method choice, is the ‘goal of the analysis’. Though most reports included in our review presented estimations simply for surveillance purposes, studies may have other dimensions to their goal, such as early warning. Goals may also vary depending on a particular study question. Generally, if estimates serve to provide an early warning signal to, for instance, trigger a prompt public health response, a more sensitive seasonality estimation method may be required. This contrasts with estimates calculated to guide the implementation of future prevention measures, where the definition of the epidemic period may need to be more stringent. This stringent definition would ensure, for example, the implementation of a cost-effective vaccination campaign or allow the most appropriate allocation of limited resources. In this regard, it is noteworthy that the first two RSV vaccines were approved in 2023 in the US and the European Union for use in people aged over 60 years, and – only one of them – in pregnant women for passive immunisation of infants up to 6 months of age [29-31]. Here, the choice of a seasonality estimation method could in part be driven by weighing the interplay between the capture rate and the length of the season as well as certain properties of a prevention measure such as the duration of protection. A higher threshold (e.g. 10% positivity vs 3% positivity) will invariably result in a shorter season, while the impact of seasonality estimation methods other than percentage positive threshold on the duration, as well as the start, end and capture rate is uncertain [13]. 

If, on the other hand, the goal of the analysis is to compare seasonality estimates emerging from different surveillance structures (e.g. multi-country or regional datasets), other elements will need to be considered as these may affect the feasibility – and the comparability – of seasonality estimates and it may be better to opt for a method which relies on the national average or expected RSV activity (category II (MEM), III (mean positivity threshold), VI−VIII (percentage, or mean, of detections threshold and AAP); (Table 2)). Doing so would result in working with a country and season specific threshold instead of a constant threshold (e.g. 10% positivity rate) applied to each national database. This, in turn, could reduce some of the background noise caused by relying on data from diverse surveillance systems.

The ‘time frame’ is another important factor to be considered as some of the methods can only be applied retrospectively (e.g. 1.2% and 60% thresholds).

The heterogeneity that exists in the ‘assessment’ characteristics (e.g. diagnostic practices, setting in which data were collected and availability of year-round data) will also affect the methods that can be applied (e.g. PCR diagnostic which may require another method than the 10% positivity threshold; RS10, which can only be applied with year-round data) and seasonality estimates. In terms of interpreting seasonality estimates, countries that limit their surveillance to the winter period rather than apply year-round surveillance may miss out on deviations from the ‘typical’ RSV season, as observed during the COVID-19 pandemic [15]. In this respect, the availability of year-round data was recommended in a 2021 report on RSV surveillance in Europe [11]. 

Experience from the COVID-19 pandemic has shown that the stability of testing practices to detect people with a given viral infection is an important factor regarding the reliability of either related case counts or positivity rates [32] and this has an impact on seasonality estimates and their interpretation. Indeed, solely relying on case counts can provide an incorrect picture, as case counts usually represent only a fraction of the actual number of infections and are highly dependent on testing practices. If the number of tests that are conducted changes over time this could result in either an increase or decrease in the number of cases that are found. In these instances, it might be best to rely on a method that corrects for this by relying on the number of specimens tested. However, changes in testing practices (e.g. diagnostics, changing sampled population) may consequentially also result in a misleading picture of the positivity rate.

Another important consideration related to ‘assessment’ characteristics is the RSV case definition used, which varied across the studies included in our review. According to the WHO, the recommended case definitions for RSV are ARI in the community setting and extended SARI in the hospitalised setting [3], with several studies showing that these are most sensitive in capturing RSV cases [4,5]. Albeit sometimes included if a study used a mix of case definitions, none of the studies we identified in the literature review used the extended SARI case definition exclusively, and only one used the ARI case definition exclusively. Though limited literature exists on the impact that these factors have on surveillance data or seasonality estimates, they could be of importance and should be evaluated.

The elements presented under ‘healthcare system and policies’ and other ‘contextual’ factors cover the data collection setting, available monitoring conditions and climatological characteristics that can differ sub-nationally, nationally and regionally. These elements are related to health expenditure and the nature and accessibility of the healthcare system (e.g. primary and community care, hospitals) in which surveillance is embedded (‘healthcare system and policies’) as well as the population’s geographical, demographical and socioeconomical context (‘contextual’ factors). They can be considered when interpreting seasonal estimates. For example, a publication with data from countries around the world reported that the age of the patients with RSV attending community care was higher than in hospitalised care, which may relate to a higher risk of severe infection in children, but could also be due to the differences in the population being sampled in both settings [33]. In addition, for influenza, it has been shown that the quality of surveillance data is associated with a higher number of reporting facilities and greater health expenditure [34]. The elements under ‘healthcare system and policies’ can moreover be connected. Access to healthcare, for instance, is a known component of a set of national context elements like public and private health expenditure, and these vary strongly between countries [35,36]. Within countries, access to healthcare is often negatively associated with socioeconomic status (SES), an established risk factor for RSV hospitalisation [9,10]. A surveillance system that (partially) excludes or misses certain populations may result in a skewed picture of the epidemic, and thus affect the seasonality estimates. These elements could even end up impacting the choice of an estimation method if further research shows them to affect seasonality estimates in a certain direction.

While other researchers have previously attempted to compare methods for the estimation of RSV seasonality [34], our study is, to the best of our knowledge, the first review that attempted to systematically cover all methods that have been used for seasonality estimation in recent years. Its strength lies in the summary and categorisation of these methods and the description of factors, including assessment characteristics, that underlie the analyses. Further to this, the potential consequences of these characteristics on seasonality estimates is thoroughly discussed. A few of the methods that we retained and described have limitations that may discourage their use (e.g. 10% positivity threshold in the case of PCR testing), but we are confident of the value added by our detailed examination for those interested in RSV seasonality. Though some kinds of seasonality analyses (e.g. transmission modelling) were not specifically included in this review, the factors identified (e.g. diagnostics) are also relevant for these and have proven relevant in other types of analyses (e.g. burden of disease studies) [37]. 

Nevertheless, our review also has some limitations, one of which is the relatively narrow scope of our search. Our search was conducted using two databases and included articles published between 2016 and 2021. In addition, no grey literature was searched. We checked all included papers for relevant references, but it is conceivable that we missed some less commonly used methods. It is also important to note that methods to define seasonality continue to evolve. However, since the aim of the study was to provide a general overview of common seasonality estimation methods and assist in the choice and interpretation of estimation methods, we felt the current strategy suffices. Another limitation is the lack of literature stemming from low- and middle-income countries (LMICs), some of which are located in regions that often experience less clear, unstable, RSV seasonality compared with temperate climates. However, one of two studies published in 2021 on RSV seasonality found that 75% of included LMICs (39/52) experienced ‘clear’ (defined as 75% of RSV cases occurring in a period of ≤ 5 months) seasonality meaning that they are likely to benefit from applying these methods, albeit perhaps more regularly as season-to-season variations are likely to exist [38,39]. 

Though we believe our categorisation of methods as well as considerations in choosing a method to be relevant for all contexts, it is not straightforward what methods are best suited for given contexts. This relates to the last limitation we want to acknowledge, namely that we did not test the relative impact of the eight categories of methods on the definition of an RSV epidemic, as well as the relative importance of underlying assessment characteristics and different types of seasonality in the current paper. While this would constitute a natural continuation and completion of the systematic review that we conducted and presented, we felt that applying the different methods to RSV surveillance data, conducting a comparative analysis, and describing the advantages and disadvantages of each method would deserve a separate dedicated work, which we plan for the near future.

### Conclusions

Our review showed how a wide range of seasonality estimation methods is currently being applied to estimate the start, end and duration of RSV epidemics. This diversity, in combination with a heterogeneous application, challenges the interpretability of results as well as comparability of RSV seasonality estimates across countries and the scientific literature. We were able to outline several discrepancies in the assessment characteristics that underlie recent seasonality estimates and discuss their (potential) implications for choosing a seasonality estimation method as well as interpreting results. However, further research should be initiated to evaluate these characteristics’ impact on RSV estimates of seasonality and their relative importance. In situations where time sensitive and costly prevention methods are being applied, providing adequate seasonality estimates is critical. Our categorisation of seasonality methods, as well as the synthesis of factors of importance, helps with both the interpretability of results and assists to some extent in the choice of an appropriate seasonality estimation method depending on a study aim and circumstances. Finally, our categorisation of methods and framework of factors has applications to defining the seasonality of other respiratory infections, for example those caused by influenza virus and SARS-CoV-2.

## Supplementary Data

313000 Supplementary Material
