# Supplementary material for: Determining the timing of respiratory syncytial virus (RSV) epidemics: a systematic review, 2016 to 2021; method categorisation and identification of influencing factors
Source: Euro Surveill. 2024 Feb 1;29(5):2300244. doi: 10.2807/1560-7917.ES.2024.29.5.2300244 (PMC10835753; doi:10.2807/1560-7917.ES.2024.29.5.2300244)
Supplement: Supplementary Material [file 2300244_SupplementaryMaterial.pdf]

This supplementary material is hosted by Eurosurveillance as supporting information alongside the article “*Determining the timing of respiratory syncytial virus (RSV) epidemics: a systematic review, 2016 to 2021; method categorisation and identification of influencing factors”*, on behalf of the authors, who remain responsible for the accuracy and appropriateness of the content. The same standards for ethics, copyright, attributions and permissions as for the article apply. Supplements are not edited by Eurosurveillance and the journal is not responsible for the maintenance of any links or email addresses provided therein.

Supplement

Search terms used in PubMed

((RSV) OR (Respiratory Syncytial Virus) OR ‘rsv’) AND ((Season*) OR (Timing) OR (Temporal) OR (Periodicity))

Search terms used in Embase

('rsv' OR 'respiratory syncytial virus'/exp) AND (season* OR 'timing'/exp OR temporal OR 'periodicity'/exp)

Supplement Table 1: study characteristics of included studies categorized by method description.

Author, year	Level	Country/Region	Subnational	Data collection purpose	Timeframe	# of cases/% positive	# of gap weeks	Computation method	Year round data collection	Age category	Setting	Case definition	Diagnostics	Category	Method description	Minimum testing	Start season definition	End season definition
Grilc et al., 2021[1]	National	Slovenia	-	Surveillance	Retrospective	% positive	0	-	Yes	All ages	Hospital data & primary care data	NA	PCR	% positivity threshold	3% positivity threshold	20 specimens	First of 2 consecutive weeks when percentage of tests positive for RSV is>3%	Last week that percentage of tests positive for RSV is>3%
Ambrose et al., 2019[2]	National	USA	-	Surveillance	Retrospective	% positive	NA	-	Yes	All ages	Hospital data (inpatient and/or outpatient)	NA	PCR			11 specimens		
Midgley et al., 2017[3]	National	USA	-	Surveillance	Retrospective	% positive	NA	-	NA	All ages	NA	NA	PCR			NA		

Olajide et al., 2018[4]	National	USA	-	Surveillance	Retrospective	% positive	NA	-	Yes	NA	Hospital	NA	PCR			NA		Last of 2 consecutive weeks when the mean percentage of positive specimens was 3%
Grilc et al., 2021[1]	National	Slovenia	-	Surveillance	Retrospective	% positive	0	-	Yes	All ages	Hospital data & primary care data	NA	PCR		10% positivity threshold	20 specimens	First of 2 consecutive weeks where => 10% of samples tested positive for RSV	Last of 2 consecutive weeks where => 10% of samples tested positive for RSV
Servia-Dopazo et al., 2020[5]	Subnational	Spain	Galicia	Surveillance	Retrospective	% positive	NA	-	Yes	Children	Hospital: in and outpatient	Bronchiolitis	Mix					
Chadha et al., 2020 [6]	Regional	Global	-	Surveillance	Retrospective	% positive	1	-	Yes (except: Canada)	All ages	Inpatient & Outpatient	Extended SARI/ARI	PCR					
Ambrose et al., 2019 [2]	National	USA	-	Surveillance	Retrospective	% positive	NA	-	Yes	All ages	Hospital data (inpatient and/or outpatient?)	NA	Antigen			11 specimens		
Ambrose et al., 2019 [2]	National	USA	-	Surveillance	Retrospective	% positive	NA	-	Yes	All ages	Hospital data (inpatient and/or outpatient?)	NA	PCR					
Ambrose et al., 2019 [2]	National	USA	-	Surveillance	Retrospective	% positive	NA	-	Yes	All ages	Hospital data (inpatient and/or outpatient?)	NA	Mix					
Gentile et al., 2019 [7]	Subnational	Argentina	Buenos Aires	Study	Retrospective	% positive	NA	-	NA	Children	Hospital: Inpatient	ALRI acquired in the community	Mix			2 specimens		
Gentile et al., 2020 [8]	National	Argentina	-	Surveillance	Realtime	% positive	1	-	Yes	Children	Hospital: Inpatient	ALRTI acquired in the community	Mix					

Glick et al., 2017 [9]	National	USA	-	Surveillance	Retrospective	% positive	1	-	No	All ages	NA	NA	Antigen			NA		
Haynes et al., 2016 [10]	National	USA	-	Surveillance	Retrospective	% positive	NA	-	No	All ages	Mix	NA	Antigen					
Midgley et al., 2017 [3]	National	USA	-	Surveillance	Retrospective	% positive	NA	-	NA	All ages	NA	NA	Antigen					
Obando-Pacheco et al., 2018 [11]	Regional	Global	-	Review	Retrospective	% positive	1	-	NA	NA	NA	NA	unknown					
Ramaekers et al., 2017 [12]	Subnational	Belgium	Leuven	Hospital database	Retrospective	% positive	NA	-	Yes	All ages	Hospital: Inpatient	ARI	PCR					
Reis et al., 2019 [13]	National	USA	-	Surveillance	Prospective	% positive	NA	-	NA	All ages	NA	NA	Mix					NA
Reis et al., 2019 [13]	National	USA	-	Surveillance	Prospective	% positive	NA	-	NA	All ages	NA	NA	Mix					
Reis et al., 2019 [13]	National	USA	-	Surveillance	Prospective	% positive	NA	-	NA	All ages	NA	NA	Mix					
Pellegrinelli et al., 2020 [14]	Subnational	Italy	Lombardy	Surveillance	Retrospective	% positive	1	-	No	All ages	Ambulatory facilities (pediatricians and general practitioners)	ILI	PCR				First week when the % of cases testing positive for RSV exceeded the 10% threshold	
Yu et al. , 2019 [15]	Subnational	China	Beijing	Study	Retrospective	% positive	0	Logistic regression model	Yes	Children	Hospital: Inpatient	Pneumonia (symptoms listed)	PCR					Last week when the % of cases testing positive exceeded the 10% threshold
Ambrose et al., 2019 [2]	National	USA	-	Surveillance	Retrospective	% positive	NA	-	Yes	All ages	Hospital data (inpatient and/or outpatient ?)	NA	Mix		3 & 10% positivity threshold	11 specimens	First of 2 consecutive weeks when positivity above threshold	Last of 2 consecutive weeks when positivity above threshold

Grilc et al., 2021[1]	National	Slovenia	-	Surveillance	Retrospective	% positive	0	-	Yes	All ages	Hospital data & primary care data	NA	PCR		5% positivity threshold	20 specimens		
Grilc et al., 2021 [1]	National	Slovenia	-	Surveillance	Retrospective	% positive	0	-	Yes	All ages	Hospital data & primary care data	NA	PCR		7% positivity threshold	20 specimens		
Baumeister et al., 2019 [16]	National	Argentina	-	Surveillance	Retrospective	% positive	NA	-	Yes	All ages	Health Care sites	Suspected of respiratory ilness (subset uses ILI and SARI others outbreak response and physicians orders)	Antigen			NA	% positive higher than annual mean percentage for two consecutive weeks	% positive below the annual mean percentage for two consecutive weeks
Rose et al., 2021 [17]	Subnational	Kenya	Siaya, -irobi, Kilifi County	Surveillance	Retrospective	% positive	2	5 week moving average	Yes	All ages	Hospital: inpatient & outpatient	Mix (ILI, ALRI, SARI)	Mix	**Mean positivity threshold**	NA	NA	The first of 3 consecutive weeks during which the moving average percentage positive was greater than the mean of the 5 week moving average percentage positive for that calendar year	The third of 3 consecutive weeks at least 5 weeks following season onset during which the moving average percentage positive was lower than the mean of the 5 week moving average percentage positive for that calendar year

Chi et al., 2018[18]	National	Taiwan	-	Claims database	Retrospective	% positive	NA	-	Yes	Children	Hospital admissions	ICD codes for RSV	NA			NA	The RSV season was defined as RSV rate in two or more consecutive months that was above the baseline rate.	The RSV season was defined as RSV rate in two or more consecutive months that was above the baseline rate.
Midgley et al., 2017 [3]	National	USA	-	Surveillance	Retrospective	# of cases	NA	4 week moving average	NA	All ages	NA	NA	PCR	**Number of detections threshold**	10-fold baseline	NA	First of 2 consecutive weeks when RSV detections are >10 times the preseason baseline	Last week when RSV detections are >10 times the preseason baseline
Vos et al., 2019 [19]	National	Netherlands	-	Surveillance	Retrospective	# of cases	NA	-	Yes	All ages	GP, hospital, outpatient clinics	ILI in primary care and unknown for virological surveillance	PCR		# of case threshold	NA	First week when more than 20 detections are registered	Last week when more than 20 detections were registered
Baker et al. , 2019 [20]	National	USA & Mexico	-	Hospital database	Retrospective	incidence	NA	Transmission model	NA	NA	Hospital: Inpatient	ICD-9 codes RSV	NA			NA	Normalized incidence (based on the mean over several years) exceeds 0.2	NA

Rose et al., 2018 [21]	National	USA	-	Surveillance	Retrospective	# of cases	0	5 week moving average	Yes	NA	Mix	NA	PCR		Retrospective slope 10 (RS10)	NA	The 2nd of 2 consecutive weeks when the slope exceeded 10 normalized detections per week (provided that the slope exceed 10 from that week forward)	Last week that normalized detections per week exceeded 10
Midgley et al., 2017 [3]	National	USA	-	Surveillance	Retrospective	# of cases	NA		NA	All ages	NA	NA	PCR			NA		
Grilc et al., 2021 [1]	National	Slovenia	-	Surveillance	Retrospective	% positive	0	-	Yes	All ages	Hospital data & primary care data	NA	PCR	**Moving epidemic Method (MEM)**	NA	NA	First week when curve exceeds the epidemic threshold (based on historical surveillance data)	First week when curve is below the post-epidemic threshold (based on historical surveillance data)
Harcourt et al., 2019 [22]	National	England	-	Surveillance	Realtime	% consultations	0	7 day moving average	Yes	Children	GP out of hours and NHS 111 calls	syndromic indicators (cough or bronchitis)	NA					
Vos et al., 2019 [19]	National	Netherlands	-	Surveillance	Retrospective	# of cases	NA	-	Yes	All ages	GP, hospital, outpatient clinics	ILI in primary care and unknown for virological surveillance	PCR					
Callahan et al., 2020 [23]	Subnational	USA	Utah	Surveillance	Retrospective	# of cases	NA	Wavelet transform	Yes	NA	Hospital & clinics	NA	Mix	**Change point analysis**	NA	NA	No formal definition, modelled via change point analysis	No formal definition, modelled via change point analysis
Broberg et al., 2018 [24]	Regional	Europe	-	Surveillance	Retrospective	# of cases	1	-	NA	NA	NA	Mix (ILI, ARI, diagnostic need)	Mix	**% of cases**	1.2% of total detections (weekly)	NA	First week RSV detections exceed 1.2%	Last week RSV detections exceed 1.2%

Grilc et al., 2021 [1]	National	Slovenia	-	Surveillance	Retrospective	# of cases	1	-	Yes	All ages	Hospital data & primary care data	NA	PCR				of total RSV positive specimens	of total RSV positive specimens
Vos et al., 2019 [19]	National	Netherlands	-	Surveillance	Retrospective	# of cases	NA	-	Yes	All ages	GP, hospital, outpatient clinics	ILI in primary care and unknown for virological surveillance	PCR					
Broberg et al., 2016 [25]	Regional	Europe	-	Surveillance	Retrospective	# of cases	NA	-	NA	NA	primary care and hospital care	NA	NA		5% of total detections (weekly)	NA	First week when the number of detections exceeds 5% of total detections	First week when detections no longer exceed 5% of total detections
Paz-Bailey et al., 2018 [26]	Subnational	Puerto Rico	Ponce and Guayama (Southern Puerto Rico)	Surveillance	Retrospective	# of cases	NA	-	NA	NA	Emergency department	onset of fever ≤7 days of presentation	PCR		10% of total detections (monthly)	NA	Month containing 10% of total annual cases for 2 years or more	-
Shapiro et al., 2017 [27]	Subnational	Sri Lanka	Southern Sri Lanka	Study	Retrospective	# of cases	NA	-	Yes	All ages	Hospital: outpatient	ILI	PCR			NA	The monthly proportion being => 10% of total cases	The monthly proportion being => 10% of total cases
Wrotek et al., 2020 [28]	Subnational	Poland	Warsaw	Study	Retrospective	# of cases	NA	-	Yes	Children	Hospital: Inpatient	Sings and symptoms indicating the involvement of the lower	PCR		2% of total detections (weekly)/8.5% of total detections (monthly)	NA	Epidemic week => 2% of total RSV cases, epidemic month => 8.5% of total RSV cases.	NA

												respiratory tract						
Montgomery et al., 2021[29]	Subnational	USA	Hawaii	Hospital database	Retrospective	# of cases	NA	-	Yes	Children	NA	NA	Mix		Mean threshold	NA	Above the average monthly number of cases	Above the average monthly number of cases
Glatman-Freedman et al., 2020 [30]	National	Israel	-	Hospital database	Retrospective	# of hospitalizations	NA	-	Yes	Children	Hospital: Inpatient	ICD codes for RSV	NA	**Mean detections threshold**	Mean plus 2SD	NA	Number of monthly hospitalizations exceeds the baseline (mean) plus 2SD	Number of monthly hospitalizations falls below baseline (mean) +2SD
Ferrero et al., 2016 [31]	Subnational	Argentina	Buenos Aires	Hospital database	Retrospective	# of cases	0	-	Yes	Children	Hospital data (inpatient and/or outpatient)	NA	NA		60% threshold	NA	First week when the number of RSV cases identified is above 60% from the average weekly identifications for that year	The first week when the number of RSV cases identified is below 60% from the average weekly cases for that year
Grilc et al., 2021 [1]	National	Slovenia	-	Surveillance	Retrospective	# of cases	0	-	Yes	All ages	Hospital data & primary care data	NA	PCR			NA		
Li et al., 2019 [32]	Regional	Global	-	Review	Retrospective	# of cases	0	-	NA	NA	NA	NA	NA	**Average Annual Percentage (AAP)**	AAP	NA	First month of the longest consecutive months to be included in the sorted AAP 75%	Last month of the longest consecutive months to be included in the sorted AAP 75%

Bibliography

1. Grilc E, Prosenc Trilar K, Lajovic J, Sočan M. Determining the seasonality of respiratory syncytial virus in Slovenia. Influenza Other Respir Viruses. 2021;15(1):56-63. http://dx.doi.org/10.1111/irv.12779 PMID:32656961

2. Ambrose CS, Steed LL, Brandon M, Frye K, Olajide IR, Thomson G. National and regional modeling of distinct RSV seasonality thresholds for antigen and PCR testing in the United States. J Clin Virol. 2019;120:68-77. http://dx.doi.org/10.1016/j.jcv.2019.09.010 PMID:31590113

3. Midgley CM, Haynes AK, Baumgardner JL, Chommanard C, Demas SW, Prill MM, et al. Determining the seasonality of respiratory syncytial virus in the United States: The impact of increased molecular testing. J Infect Dis. 2017;216(3):345-55. http://dx.doi.org/10.1093/infdis/jix275 PMID:28859428

4. Olajide I, Ambrose C, Dube C. New method for determining respiratory syncytial virus seasonality in the united states: Results from RSV alert, a hospital-based laboratory surveillance program. J Manag Care Spec Pharm . 2018;24(10 A):S67.

5. Servia-Dopazo M, Purriños-Hermida MJ, Pérez S, García J, Malvar-Pintos A, et al. [Usefulness of the microbiological surveillance of respiratory syncytial virus in Galicia (Spain): 2008-2017]. Gac Sanit. 2020;34(5):474-9. http://dx.doi.org/10.1016/j.gaceta.2018.11.009 PMID:30737055

6. Chadha M, Hirve S, Bancej C, Barr I, Baumeister E, Caetano B, et al.; WHO RSV Surveillance Group. Human respiratory syncytial virus and influenza seasonality patterns-Early findings from the WHO global respiratory syncytial virus surveillance. Influenza Other Respir Viruses. 2020;14(6):638-46. http://dx.doi.org/10.1111/irv.12726 PMID:32163226

7. Gentile A, Lucion MF, Juarez MDV, Areso MS, Bakir J, Viegas M, et al. Burden of Respiratory Syncytial Virus Disease and Mortality Risk Factors in Argentina: 18 Years of Active Surveillance in a Children's Hospital. Pediatr Infect Dis J. 2019;38(6):589-94. http://dx.doi.org/10.1097/INF.0000000000002271 PMID:30672892

8. Gentile Á, Lucion MF, Juárez MDV, Castellano V, Bakir J, Pacchiotti A, et al. Virus sincicial respiratorio en niños nacidos prematuros: 19 años de vigilancia epidemiológica activa en un hospital pediátrico. [Respiratory syncytial virus in preterm infants: 19 years of active epidemiological surveillance in a children's hospital]. Arch Argent Pediatr. 2020;118(6):386-92. PMID:33231045

9. Glick AF, Kjelleren S, Hofstetter AM, Subramony A. RSV Hospitalizations in Comparison With Regional RSV Activity and Inpatient Palivizumab Administration, 2010-2013. Hosp Pediatr. 2017;7(5):271-8. http://dx.doi.org/10.1542/hpeds.2016-0124 PMID:28381595

10. Haynes AK, Fowlkes AL, Schneider E, Mutuc JD, Armstrong GL, Gerber SI. Human Metapneumovirus Circulation in the United States, 2008 to 2014. Pediatrics. 2016;137(5):e20152927. http://dx.doi.org/10.1542/peds.2015-2927 PMID:27244790

11. Obando-Pacheco P, Justicia-Grande AJ, Rivero-Calle I, Rodríguez-Tenreiro C, Sly P, Ramilo O, et al. Respiratory Syncytial Virus Seasonality: A Global Overview. J Infect Dis. 2018;217(9):1356-64. http://dx.doi.org/10.1093/infdis/jiy056 PMID:29390105

12. Ramaekers K, Keyaerts E, Rector A, Borremans A, Beuselinck K, Lagrou K, et al. Prevalence and seasonality of six respiratory viruses during five consecutive epidemic seasons in Belgium. J Clin Virol. 2017;94:72-8. http://dx.doi.org/10.1016/j.jcv.2017.07.011 PMID:28772168

13. Reis J, Yamana T, Kandula S, Shaman J. Superensemble forecast of respiratory syncytial virus outbreaks at national, regional, and state levels in the United States. Epidemics. 2019;26:1-8. http://dx.doi.org/10.1016/j.epidem.2018.07.001 PMID:30025885

14. Pellegrinelli L, Galli C, Bubba L, Cereda D, Anselmi G, Binda S, et al. Respiratory syncytial virus in influenza-like illness cases: Epidemiology and molecular analyses of four consecutive winter seasons (2014-2015/2017-2018) in Lombardy (Northern Italy). J Med Virol. 2020;92(12):2999-3006. http://dx.doi.org/10.1002/jmv.25917 PMID:32314816

15. . Yu J, Liu C, Xiao Y, Xiang Z, Zhou H, Chen L, et al. Respiratory Syncytial Virus Seasonality, Beijing, China, 2007-2015. Emerg Infect Dis. 2019;25(6):1127-35. http://dx.doi.org/10.3201/eid2506.180532 PMID:31107230

16. Baumeister E, Duque J, Varela T, Palekar R, Couto P, Savy V, et al. Timing of respiratory syncytial virus and influenza epidemic activity in five regions of Argentina, 2007-2016. Influenza Other Respir Viruses. 2019;13(1):10-7. http://dx.doi.org/10.1111/irv.12596 PMID:30051595

17. Rose EB, Nyawanda BO, Munywoki PK, Murunga N, Bigogo GM, Otieno NA, et al. Respiratory syncytial virus seasonality in three epidemiological zones of Kenya. Influenza Other Respir Viruses. 2021;15(2):195-201. http://dx.doi.org/10.1111/irv.12810 PMID:33305543

18. Chi H, Chung C-H, Lin Y-J, Lin C-H. Seasonal peaks and risk factors of respiratory syncytial virus infections related hospitalization of preterm infants in Taiwan. PLoS One. 2018;13(5):e0197410. http://dx.doi.org/10.1371/journal.pone.0197410 PMID:29746578

19. Vos LM, Teirlinck AC, Lozano JE, Vega T, Donker GA, Hoepelman AI, et al. Use of the moving epidemic method (MEM) to assess national surveillance data for respiratory syncytial virus (RSV) in the Netherlands, 2005 to 2017. Euro Surveill. 2019;24(20):1800469. http://dx.doi.org/10.2807/1560-7917.ES.2019.24.20.1800469 PMID:31115311

20. Baker RE, Mahmud AS, Wagner CE, Yang W, Pitzer VE, Viboud C, et al. Epidemic dynamics of respiratory syncytial virus in current and future climates. Nat Commun. 2019;10(1):5512. http://dx.doi.org/10.1038/s41467-019-13562-y PMID:31797866

21. Rose EB, Wheatley A, Langley G, Gerber S, Haynes A. Respiratory Syncytial Virus Seasonality - United States, 2014-2017. MMWR Morb Mortal Wkly Rep. 2018;67(2):71-6. http://dx.doi.org/10.15585/mmwr.mm6702a4 PMID:29346336

22. Harcourt SE, Morbey RA, Smith GE, Loveridge P, Green HK, Pebody R, et al. Developing influenza and respiratory syncytial virus activity thresholds for syndromic surveillance in England. Epidemiol Infect. 2019;147:e163. http://dx.doi.org/10.1017/S0950268819000542 PMID:31063101

23. Callahan ZY, Smith TK, Ingersoll C, Gardner R, Korgenski EK, Sloan CD. Comparative Seasonal Respiratory Virus Epidemic Timing in Utah. Viruses. 2020;12(3):275. http://dx.doi.org/10.3390/v12030275 PMID:32121465

24. Broberg EK, Waris M, Johansen K, Snacken R, Penttinen P, Trebbien R , et al.; European Influenza Surveillance Network. Seasonality and geographical spread of respiratory syncytial virus epidemics in 15 European countries, 2010 to 2016. Euro Surveill. 2018;23(5):17-00284. http://dx.doi.org/10.2807/1560-7917.ES.2018.23.5.17-00284 PMID:29409569

25. Broberg E, Johansen K, Adlhoch C, Snacken R, Penttinen P. Seasonality of respiratory syncytial virus infection in the EU/EEA, 2010-2016. J Clin Virol. 2016;82:S116-7. http://dx.doi.org/10.1016/j.jcv.2016.08.234

26. Paz-Bailey G, Adams L, Munoz J, Lozier M, Olsen S, Blanton L, et al. Trends in influenza and other respiratory viruses in Southern Puerto Rico, 2012-2017. Am J Trop Med Hyg. 2018;99(4):524.

27. Shapiro D, Bodinayake CK, Nagahawatte A, Devasiri V, Kurukulasooriya R, Hsiang J, et al. Burden and Seasonality of Viral Acute Respiratory Tract Infections among Outpatients in Southern Sri Lanka. Am J Trop Med Hyg. 2017;97(1):88-96. http://dx.doi.org/10.4269/ajtmh.17-0032 PMID:28719323

28. Wrotek A, Czajkowska M, Jackowska T. Seasonality of Respiratory Syncytial Virus Hospitalization. Adv Exp Med Biol. 2020;1279:93-100. http://dx.doi.org/10.1007/5584_2020_503 PMID:32170670

29. Montgomery AS, Lustik MB, Jones MU, Horseman TS. Respiratory Viral Pathogens in Children Evaluated at Military Treatment Facilities in Oahu, Hawaii From 2014 to 2018: Seasonality and Climatic Factors. J Pediatric Infect Dis Soc. 2021;10(4):517-20. http://dx.doi.org/10.1093/jpids/piaa131 PMID:33219667

30. Glatman-Freedman A, Kaufman Z, Applbaum Y, Dichtiar R, Steiman A, Gordon E-S, et al. Respiratory Syncytial Virus hospitalization burden: a nation-wide population-based analysis, 2000-2017. J Infect. 2020;81(2):297-303. http://dx.doi.org/10.1016/j.jinf.2020.05.078 PMID:32504738

31. Ferrero F, Torres F, Abrutzky R, Ossorio MF, Marcos A, Ferrario C, et al. Seasonality of respiratory syncytial virus in Buenos Aires. Relationship with global climate change. Arch Argent Pediatr. 2016;114(1):52-5. PMID:26914075

32. Li Y, Wang X, Blau DM, Caballero MT, Feikin DR, Gill CJ, et al.; Respiratory Virus Global Epidemiology Network; RESCEU investigators. Global, regional, and national disease burden estimates of acute lower respiratory infections due to respiratory syncytial virus in children younger than 5 years in 2019: a systematic analysis. Lancet. 2022;399(10340):2047-64. http://dx.doi.org/10.1016/S0140-6736(22)00478-0 PMID:35598608
